# The relationship between school belonging and depressive symptoms among Chinese college students: a moderated mediation model

**DOI:** 10.3389/fpsyg.2026.1728017

**Published:** 2026-02-05

**Authors:** Ai Yun, Yi Cai, Shuang Li, Isakova Ch. B., Guanyu Cen, Yuanyan Hu

**Affiliations:** 1 Mental Health Education and Counseling Center, Chongqing Chemical Industry Vocational College, Chongqing, China; 2 Laboratory of Emotion and Mental Health, Chongqing University of Arts and Sciences, Chongqing, China; 3 Department of Psychology, School of Education, Soochow University, Suzhou, China; 4 Institute of Education and Psychology, Kyrgyz State University, Bishkek, Kyrgyzstan; 5 K. Tynystanov Institute of Higher Education, Karakol, Kyrgyzstan; 6 Postdoctoral Workstation of Art Theory, Southeast University, Nanjing, China

**Keywords:** Chinese college students, depressive symptoms, emotional intelligence, school belonging, self-consistency and congruence

## Abstract

**Objective:**

To explore the relationship and mechanism between school belonging and depressive symptoms among Chinese college students.

**Methods:**

A total of 440 Chinese college students were surveyed using the Chinese version of the Psychological Sense of School Membership Scale (PSSM-CR), the Short Version of the Center for Epidemiologic Studies Depression Scale (CESD-10), the Self-Consistency and Congruence Scale (SCCS), the Schutte Self-report Emotional Intelligence Scale (SSEIS-C).

**Results:**

(1) School belonging, self-consistency and congruence, and depressive symptoms were significantly correlated (*r* = −0.53 to 0.74, *p* < 0.01); (2) Self-consistency and congruence played a partial mediating role in the relationship between school belonging and depressive symptoms among Chinese college students; (3) Emotional intelligence significantly moderated the first half of the mediating model: the first half of the mediating model was significant in both the low emotional intelligence group and the high emotional intelligence group.

**Conclusion:**

Self-consistency and congruence and emotional intelligence play a moderated mediation role in the relationship between school belonging and depressive symptoms among Chinese college students.

## 1 Introduction

Depression among college students has emerged as a global public health issue (Li et al., 2021). For instance, studies reveal that 39.4% of 15,859 university students across Cambodia, Laos, Malaysia, Myanmar, Thailand, and Vietnam in Southeast Asia suffer from depression (Dessauvagie et al., 2022). Similarly, research indicates that 48.14% of 2,031 American college students exhibit moderate to severe depression (Wang et al., 2020). The mental health status of Chinese students likewise gives cause for grave concern. Both the Report on National Mental Health Development in China (2021–2022) and a meta-analysis encompassing 113 studies involving 185,787 Chinese college students confirm persistently elevated depression prevalence, with a detection rate of 24.1% (Fu et al., 2021; Chen et al., 2022).

Depressive symptoms refer to negative emotional states—including dejection, sadness, and hopelessness—that frequently emerge under life stressors. These manifestations resemble clinical depression but fall below the diagnostic threshold for the disorder (Luo et al., 2025). Persistent depressive symptoms jeopardize both physical and mental well-being, not only increasing vulnerability to clinical depression (Niu et al., 2025) but also potentially causing sleep disturbances, academic impairment, and related complications (Steare et al., 2024; Luo et al., 2025; Niu et al., 2025). Consequently, investigating the contributing factors and underlying mechanisms of depressive symptoms among college students holds substantial significance.

## 2 Literature review

### 2.1 School belonging and depressive symptoms

School belonging refers to the extent to which students perceive themselves as integral members of school life and activities, feeling respected, accepted, and supported by teachers and peers. This construct reflects individuals’ school experiences (e.g., academic performance and social interactions) and the strength of their emotional connection to the school (Zhai et al., 2020). The Interpersonal Risk Model emphasizes that negative interpersonal relationships constitute significant factors in the onset, persistence, and exacerbation of depression (Hames et al., 2013). When students fail to establish healthy interpersonal relationships at school, they may experience diminished peer and teacher support, weakening their emotional bond with the institution. Consequently, this reduces their sense of school belonging and increases vulnerability to depression. For instance, Yin et al. conducted cross-lagged analyses on data from the China Education Panel Survey (CEPS) in 2013 and 2014, revealing that students with poorer interpersonal relationships exhibited lower school belonging and greater susceptibility to negative emotions such as depression (Yin et al., 2024). Therefore, this study proposes Hypothesis 1: School belonging negatively predicts depressive symptoms among college students.

### 2.2 The mediating effect of self-consistency and congruence

Self-consistency and congruence is a central concept in Rogers’ personality theory, emphasizing the alignment between the self and one’s experiences, and is regarded as a crucial indicator of an individual’s mental health (Rogers, 1959). Although the concept of self-consistency and congruence originated in Western psychology, most notably in Carl Rogers’ humanistic theory as the alignment between one’s self-concept and experiential reality (Moore and Oosthuizen, 1997), it both resonates with and diverges from understandings found in Eastern traditions. Both Eastern and Western perspectives share a foundational premise: psychological well-being arises when there is alignment among internal and external dimensions of the self. Specifically, whether in Carl Rogers’ triadic model of the real self, personal experience, and ideal self or in classical Chinese thought, such as the Confucian emphasis on self-cultivation (*xiu shen*) and the Daoist principle of “following the natural way” (*dao fa zi ran*), harmony is consistently linked to a sense of inner order, psychological integration, and adaptive functioning (He, 2024; Zhang, 2025). In both frameworks, mental health is understood as more attainable when an individual’s self-perceptions, affective experiences, and outward behaviors or social contexts are not in marked discord. However, the two traditions differ substantially in their normative values and pathways to achieving such harmony. Western humanistic psychology foregrounds individual authenticity, conceptualizing the self as autonomous and emphasizing the coherence and integrity of internal experience (Resnick et al., 2001). Psychological distress, in this view, typically stems from incongruence between the self and one’s lived experience. By contrast, traditional Chinese conceptions of self-consistency and congruence are inherently relational and ethical, situating the individual within a web of familial, social, and cosmological roles and responsibilities (Ma, 2011). Ideals such as “restraining the self and returning to ritual propriety” (*ke ji fu li*), “aligning oneself with righteousness” (*yi zhi yu bi*), and the Confucian dictum of “cultivating the self in adversity and benefiting all under heaven in times of prosperity” (*qiong ze du shan qi shen, da ze jian ji tian xia*) underscore a path to psychological equilibrium through self-discipline, moral conduct, and the conscientious fulfillment of social duties.

Accordingly, this study does not uncritically import the Western construct of congruence. Rather, it treats the underlying mechanism of internal coherence as a cross-culturally relevant psychological process, while deliberately embedding it within the distinctively Chinese, relationally oriented, and ethically grounded framework of harmony. This integrative approach aims to yield a more culturally sensitive and theoretically nuanced account of the psychological mechanisms underlying depressive symptoms among Chinese college students.

College students exhibiting high levels of self-consistency and congruence demonstrate enhanced capacity to integrate internal and external resources, enabling them to mitigate or self-resolve depressive affect stemming from environmental stressors (Luo et al., 2023; Han et al., 2025). Empirical evidence confirms that college students with elevated self-consistency and congruence show significantly reduced likelihood of developing depressive symptoms (Li et al., 2014). Additionally, the Process-Person-Context-Time (PPCT) model emphasizes that individual development is significantly shaped by proximal environmental influences (Tudge et al., 2009). Unlike the commuter systems predominant in Western universities, Chinese undergraduates reside on campus for over 8 months annually. Consequently, their physical and psychological well-being exhibits heightened susceptibility to university environmental factors compared to familial or societal impacts. When institutional support proves inadequate, students manifest: diminished school belonging, intensified loneliness, fragmentation of self-perception, and incongruence between self-concept and lived experiences. These manifestations collectively constitute a state of self-inconsistency and incongruence (Li and Li, 2024; Chen C. et al., 2025). Prolonged exposure to such self-inconsistency and incongruence predisposes individuals to anxiety, depression, and related negative affective states, ultimately exacerbating depressive symptomatology (Rachel et al., 2016; Burdette et al., 2021). Therefore, this study proposes Hypothesis 2: Self-consistency and congruence mediates the relationship between school belonging and depressive symptoms among Chinese college students. Specifically, individuals with higher emotional intelligence are expected to exhibit a stronger negative association between low school belonging and self-consistency and congruence. Heightened emotional awareness may render these individuals more sensitive to the cognitive and affective consequences of belonging deficits, thereby exacerbating internal incongruence.

### 2.3 The moderating effect of emotional intelligence

Emotional intelligence was first proposed by Salovey and Mayer, encompassing three abilities: the ability to recognize the meaning of emotions and their relationships, the ability to reason and solve problems using emotion-related knowledge, and the ability to use emotions to facilitate cognitive activities (Mayer et al., 2000). According to this definition, for college students with high emotional intelligence, when they experience internal psychological conflicts due to a low sense of school belonging, they can perceive negative emotions such as loneliness arising in this process, recognize the conflict between these emotions and their self-cognition, and further transform the crisis of insufficient school belonging into an opportunity for self-growth by regulating negative emotions. This helps improve their low level of self-consistency and congruence (i.e., alleviate self-incongruence) (Gürcan-Yıldırım and Gençöz, 2022; Li et al., 2024). In contrast, college students with low emotional intelligence may be unable to correctly identify, understand, and regulate negative emotions, leading them to fall into self-deprecation or avoidance behaviors, which exacerbates self-incongruence (He et al., 2013). Empirical studies have shown that emotional intelligence significantly and negatively predicts the level of s self-incongruence (He et al., 2012; Li et al., 2024). To summarize, emotional intelligence may play a mitigating role in the tense relationship between school belonging and self-incongruence. Specifically, individuals with high emotional intelligence can reduce self-incongruence caused by insufficient school belonging through effective emotion regulation strategies such as cognitive reappraisal and problem-solving. Therefore, this study proposes Hypothesis 3: Emotional intelligence plays a moderating role in the relationship between college students’ sense of school belonging and self-consistency and congruence.

**Figure 1:**
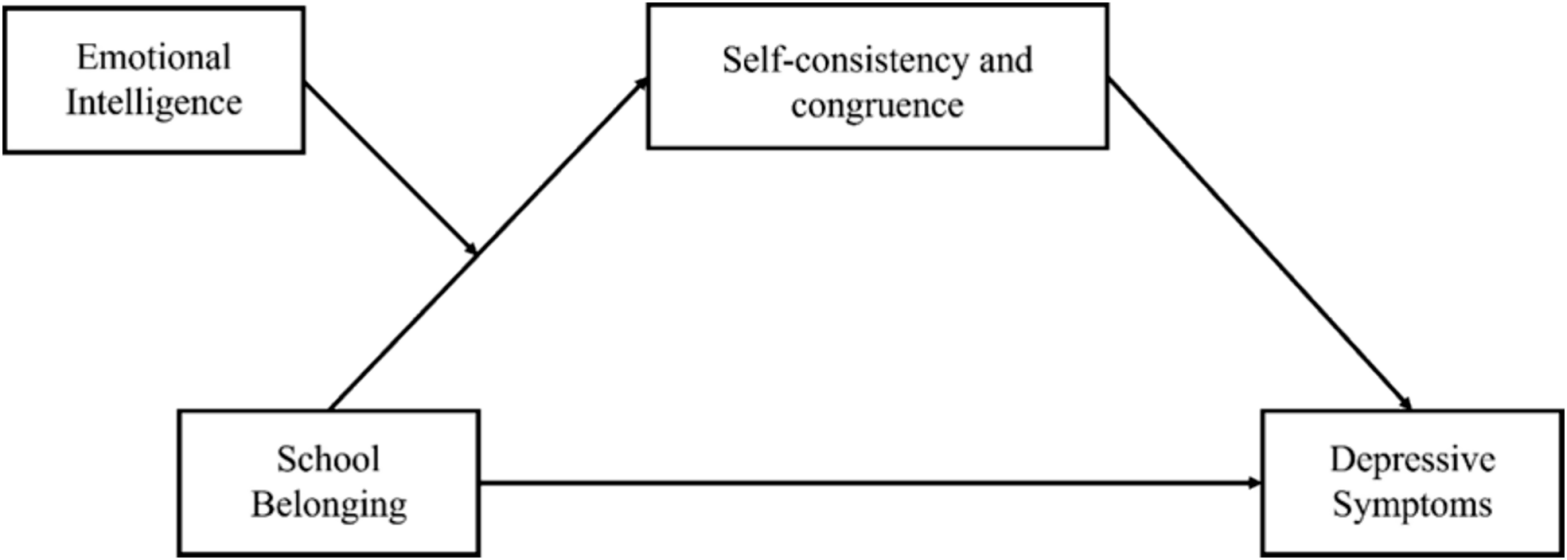
The proposed hypothesis model.

In summary, this study proposes a moderated mediation model (see Figure 1) to investigate the influencing factors and underlying mechanisms of depressive symptoms among Chinese college students, thus providing theoretical foundations for preventive interventions against depression in this population.

## 3 Methods

### 3.1 Participants

Convenience sampling was adopted to conduct a questionnaire survey among college students from three comprehensive universities in China. With the consent of the course instructors, data were collected during class breaks on a class-by-class basis. Instructors projected a QR code linking to the online survey (hosted on Wenjuanxing) onto the classroom screen, and students voluntarily completed the questionnaire on their mobile phones. Participants were informed that the survey was anonymous and voluntary, that there were no right or wrong answers, and they were encouraged to respond honestly based on their actual experiences. The average completion time was approximately 8 min.

A total of 472 students participated in the survey. After excluding responses with implausibly short completion times or clear signs of careless responding (e.g., straight-lining, repetitive patterns, or inconsistency with reverse-scored items), 440 valid questionnaires were retained for analysis, resulting in an effective response rate of 93.2%.

All participants were undergraduate students majoring in fields other than psychology and reported no history of psychiatric disorders. Among them, 45% were enrolled in humanities or social sciences programs, and 55% were in STEM fields. In terms of geographic origin, 40% were from Chongqing Municipality, 35% from Heilongjiang Province, and 25% from Liaoning Province.

The participants were aged 17–24 years, with an average age of 19.49 ± 1.66 years. Further demographic characteristics are provided in Table 1.

**Table 1:** Information of the participants.

	Gender		Grade				Place of origin	
	Male	Female	Freshman	Sophomore	Junior	Senior	Urban	Rural
*n*	175	265	195	96	92	57	253	187

### 3.2 Measurements

#### 3.2.1 The Chinese version of the Psychological Sense of School Membership Scale (PSSM-CR)

The Chinese version of the Psychological Sense of School Membership Scale (PSSM-CR) was used, which was originally developed by Goodenow (1993) and later revised by Cheung and Hui (2003). This scale consists of 18 items, including two dimensions: sense of rejection (reverse-scored; e.g., “It’s hard for people like me to be accepted here”) and sense of belonging (e.g., “I feel like a real part of this school”). A 6-point Likert scale was used for scoring, ranging from 1 (strongly disagree) to 6 (strongly agree). In this study, the Cronbach’s *α* coefficient of the PSSM-CR scale was 0.88, with higher scores indicating greater school belonging.

#### 3.2.2 The Short Version of the Center for Epidemiologic Studies Depression Scale (CESD-10)

The Short Version of the Center for Epidemiologic Studies Depression Scale (CESD-10) was used, which was originally developed by Radloff (1977) and revised by Andersen et al. (1994). This scale mainly measures the depressive status of college students, covering three dimensions: depressive emotions, positive emotions (reverse-scored), and somatic symptoms. It consists of 10 items and uses a 4-point Likert scale, ranging from 1 (none or occasionally) to 4 (all the time or always). In this study, the Cronbach’s *α* coefficient of the CESD-10 scale was 0.92, with higher scores indicating greater levels of depressive symptoms.

#### 3.2.3 Self-Consistency and Congruence Scale (SCCS)

Self-consistency and congruence were assessed using the Self-Consistency and Congruence Scale (SCCS), adapted by Wang (1994). This 35-item scale measures three dimensions: self-flexibility (reverse-scored), discrepancy between self and experience, and self-rigidity. Responses are rated on a 5-point Likert scale from 1 (completely disagree) to 5 (completely agree). In this study, the Cronbach’s *α* coefficient of the SCCS was 0.91, with higher scores indicating greater self-incongruence.

#### 3.2.4 The Schutte Self-report Emotional Intelligence Scale (SSEIS-C)

The Schutte Self-report Emotional Intelligence Scale (SSEIS-C) was used to measure the emotional intelligence of college students. This scale was originally developed by Schutte et al. (1998) and revised by Liu (2008). It covers 5 dimensions (regulation of others’ emotions, evaluation of others’ emotions, evaluation of one’s own emotions, regulation of one’s own emotions, and use of emotions) and consists of 21 items. A 5-point Likert scale is adopted for scoring, ranging from 1 (completely inconsistent) to 5 (completely consistent). In this study, the Cronbach’s *α* coefficient of the SSEIS-C was 0.95 with higher scores indicating greater emotional intelligence.

### 3.3 Statistical methods

This study mainly used SPSS 23.0 for descriptive statistical analysis of the data, and the PROCESS macro in SPSS 23.0 to test the moderated mediation model.

## 4 Results

### 4.1 Common method bias test

This study adopted Harman’s single-factor test to examine common method bias. The results of unrotated principal component factor analysis showed that there were 15 factors with eigenvalues greater than 1, and the variance explained by the first factor was 27.22%. This meets the critical criterion of being less than 40% (Zhou and Long, 2004), indicating that there is no serious common method bias in this study.

### 4.2 Descriptive statistics

As shown in Table 2, the results of descriptive statistical analysis indicated the following: College students’ sense of school belonging was significantly negatively correlated with self-consistency and congruence and depression; Emotional intelligence was significantly negatively correlated with self-consistency and congruence and depressive symptoms; College students’ sense of school belonging was significantly positively correlated with emotional intelligence; Self-consistency and congruence was significantly positively correlated with depressive symptoms.

**Table 2:** Descriptive statistics of and correlation between the investigated variables.

Variable	*M* ± *SD*	1	2	3	4
1. School belonging	4.92 ± 0.77	1			
2. Depressive symptoms	1.57 ± 0.61	−0.60^**^	1		
3. Self-consistency and congruence	2.40 ± 0.54	−0.53^**^	0.74^**^	1	
4. Emotional intelligence	4.15 ± 0.68	0.45^**^	−0.11^*^	−0.38^**^	1

^*^p < 0.05, ^**^p < 0.01. Self-consistency and congruence was scored using the original method, with higher scores indicating greater self-incongruence. The same scoring and interpretation are used in subsequent tables and figures.

### 4.3 Test of moderated mediation effect

To facilitate interpretation of effect sizes and enable comparison across coefficients, all continuous variables were standardized prior to hypothesis testing. This study used Model 7 in the PROCESS macro to test the moderated mediation model, with 5,000 repeated samplings conducted for the test. Previous research has consistently shown that depressive symptoms differ significantly by gender and grade level, and these variables are frequently included as covariates in related work to control for potential confounding effects (Chen T. et al., 2025; Liu et al., 2025). Accordingly, this study also included gender and grade as control variables in all relevant analyses. The results are shown in Table 3. The direct predictive effect of college students’ sense of school belonging on depressive symptoms was significant (*β* = −0.28, *p* < 0.001); Meanwhile, the predictive effect of college students’ sense of school belonging on self-consistency and congruence was significant (*β* = −0.35, *p* < 0.001); The predictive effect of self-consistency and congruence on depressive symptoms was significant (*β* = 0.91, *p* < 0.001); The mediating effect size of sense of school belonging on depressive symptoms through self-consistency and congruence was −0.32, with a 95% Bootstrap confidence interval of [−0.42, −0.23]. Since the interval does not contain 0, the mediating effect is significant. To this end, it can be concluded that self-consistency and congruence plays a partial mediating role in the effect of college students’ sense of school belonging on depressive symptoms.

**Table 3:** Results of moderated mediation analysis.

	Equation 1 (efficacy criterion: SCCS)			Equation 2 (efficacy criterion: CESD-10)		
	*β*	*t*	95%CI	*β*	*t*	95%CI
Gender	−0.02	−0.46	[−0.08, 0.05]	−0.06	−1.33	[−0.16, 0.03]
grade	0.04	2.34^*^	[0.01, 0.07]	0.01	0.13	[−0.04, 0.05]
PSSM-CR	−0.35	−11.36^***^	[−0.41, −0.29]	−0.28	−7.03^***^	[−0.36, −0.21]
SSEIS-C	−0.23	−8.89^***^	[−0.28, −0.18]			
PSSM-CR × SSEIS-C	−0.20	−10.40^***^	[−0.24, −0.16]			
SCCS				0.91	15.60^***^	[0.80, 1.03]
*R^2^*			0.44			0.55
*F*			67.78			132.46

*p < 0.05, ***p < 0.001. Categorical covariates were dummy-coded as follows: gender (0 = male, 1 = female); grade level (1 = Freshman, 2 = Sophomore, 3 = Junior, 4 = Senior).

Emotional intelligence significantly predicts self-consistency and congruence (*β* = −0.23, *p* < 0.001), the interaction between school belonging and emotional intelligence significantly predicted self-consistency and congruence (*β* = −0.20, *p* < 0.001), indicating that emotional intelligence plays a moderating role in the relationship between college students’ sense of school belonging and self-consistency and congruence (moderation effect: *ΔR^2^* = 0.14, *p* < 0.001), with the effect size exceeding 0.02.

**Figure 2:**
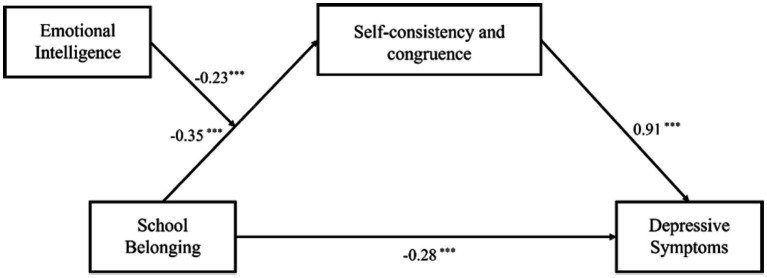
The moderated mediation model of relationship between school belonging and depressive symptoms. The path from school belonging to self-consistency and congruence is moderated by emotional intelligence (*β*_interaction_ = −0.20, *p* < 0.001), ***p < 0.001.

In summary, Chinese college students’ school belonging not only influence depressive symptoms through the mediating role of self-consistency and congruence but is also moderated by emotional intelligence in its relationship with self-consistency and congruence. The specific path coefficients are presented in Figure 2.

**Figure 3:**
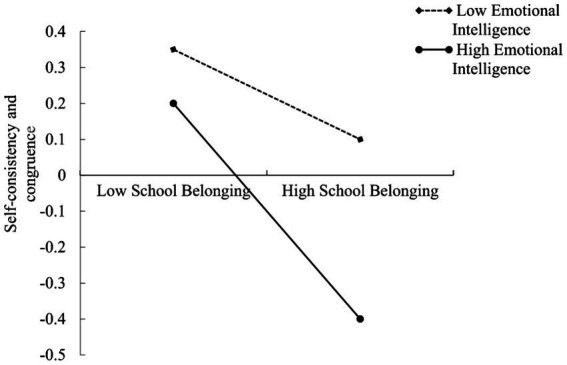
Figure of moderation effect.

To further examine how emotional intelligence moderates the relationship between school belonging and self-consistency and congruence in college students, a simple slope analysis was conducted. Emotional intelligence was divided into high or low group based on the mean plus or minus one standard deviation (Hayes and Montoya, 2016). As shown in Figure 3, when emotional intelligence is lower, school belonging still negatively predicted self-consistency and congruence, but the effect was relatively weaker (*β*_simple_ = −0.20, *t* = −6.79, *p* < 0.001). In contrast, when college students have a higher level of emotional intelligence, school belonging exists a significant negative predictive effect on self-consistency and congruence (*β*_simple_ = −0.51, *t* = −13.09, *p* < 0.001). These results suggest that higher emotional intelligence exacerbates the adverse effect of low school belonging on self-consistency and congruence.

## 5 Discussion

College students globally are a high-risk group for depression and have become a major focus in the international field of mental health (Dutcher et al., 2022). By establishing a moderated mediation model, this study explored the impact of school belonging on depressive symptoms among Chinese college students and its underlying mechanism, providing a theoretical basis and practical implications for preventing depression in college students.

### 5.1 School belonging and depressive symptoms

The results indicated that school belonging significantly and negatively predicted depressive symptoms among Chinese university students, with higher school belonging associated with lower depressive symptomatology, in line with previous research and the interpersonal risk model of depression (Hames et al., 2013; Parr et al., 2020; Yin et al., 2024). According to this framework, negative interpersonal experiences undermine individuals’ perceptions of acceptance and support, thereby reducing their sense of belonging; prolonged exposure to such relationally impoverished environments heightens vulnerability to persistent feelings of hopelessness, disappointment, and diminished self-worth, which in turn increase the likelihood of sustained depressive symptoms (Hames et al., 2013; Chen W. et al., 2025).

Notably, this association appears to be strongly amplified by the distinctive socio-ecological context of Chinese higher education. In contrast to their Western counterparts, who typically live off campus and maintain more diverse social networks, Chinese university students generally reside on campus for over 8 months per academic year, giving rise to a social ecosystem marked by high interpersonal density, sustained contact, and relative enclosure (Zhang and He, 2023). Within this context, the campus serves not only as an academic setting but also as the primary, and often sole, physical space for daily life, social interaction, and identity development. Dormitories, classes, cafeterias, and libraries constitute the core nodes of students’ social networks. The profound overlap between life space and psychological space effectively renders “school belonging” synonymous with “belonging to one’s primary life community.” Thus, the strength of students’ sense of belonging directly reflects their access to social support, relational affirmation, and emotional connectedness within their primary life domain (Li and Li, 2024). When school belonging is low, alternative buffering resources, such as sustained contact with the family of origin or integration into independent off-campus social networks, are typically unavailable, leaving individuals acutely vulnerable. The resulting frustration of the relational self consequently exerts a more direct, continuous, and destabilizing influence on self-consistency and congruence, thereby exacerbating psychological distress (Chen C. et al., 2025). Empirical observations in university settings further suggest that students’ behavioral engagement and sense of attachment may weaken even when their reported satisfaction with academic experiences remains relatively stable, underscoring the fragility of relational anchoring within institutional contexts (So et al., 2025).

Moreover, the institutionalized practice of collective living transforms the Confucian ideal of “harmony with others” from an abstract ethical norm into an everyday practical demand. Students must continuously navigate roommate dynamics, class-based group obligations, and shared living norms in close physical proximity (Han, 2008). In this intensified relational milieu, school belonging functions less as a passive affective state and more as an indicator of one’s success in integrating into a culturally esteemed relational network. High belonging signifies successful alignment with the culturally prescribed ideal of interpersonal harmony, whereas low belonging signals persistent “relational dissonance” within a core life context. Critically, such dissonance is repeatedly activated and reinforced through daily interpersonal interactions, which consistently deliver frequent and intense negative feedback to a self-construal that is fundamentally relational in nature, thereby contributing over time to the development of depressive symptoms.

Crucially, this “amplified social embeddedness” underscores a critical culture–ecology interaction. In many Western university settings, school belonging may constitute only one of several sources of social connection, with its psychological impact potentially moderated or attenuated by affiliations with family or communities (Allen et al., 2018; Hautala et al., 2022). By contrast, the institutional architecture of Chinese higher education systemically magnifies the psychological weight of school belonging. The strong predictive power of this variable in the present study thus reflects not merely a replication of Western models, but rather the emergent psychological dynamics arising from the confluence of cultural values (e.g., collectivism, relational harmony) and institutional arrangements (e.g., mandatory on-campus residence).

### 5.2 The mediating effect of self-consistency and congruence

The results also indicated that self-consistency and congruence plays a partial mediating role between college students’ sense of school belonging and depressive symptoms. Specifically, college students with a lower sense of school belonging are more likely to experience self-incongruence, which may indirectly increase their risk of developing depressive symptoms. This aligns with previous studies and can be explained by the PPCT Model (Li et al., 2014; Zhu and Li, 2016). As the proximal environment for college students, the university campus—along with its related environmental characteristics (e.g., school climate, peer and teacher support)—influences the occurrence of depressive symptoms in individuals (Tudge et al., 2009; Liu et al., 2022). If college students can obtain sufficient sense of school belonging from the university, it will help them better integrate internal self-information and external experience, improve their level of self-consistency and congruence, and tend to adopt positive self-regulation strategies to alleviate negative emotions such as anxiety and depression.

Notably, rooted in Confucian philosophy, the notion of self-consistency and congruence articulated in the Doctrine of the Mean (*Zhongyong*) emphasizes emotional regulation through “expressing feelings in due measure” (*fa er jie zhong jie*) and the attainment of dynamic equilibrium between inner experience and outward conduct (*zhi zhong he*), a conception that aligns closely with core features of the Chinese cultural schema (Yang, 2009). Although this conception is functionally comparable to Rogersian congruence, it places greater emphasis on relational embeddedness and role-based obligations (Ma, 2011). Specifically, psychological distress among Chinese college students often stems less from Rogersian “blocked self-actualization” and more from conflicts between competing role expectations and relational disruptions—such as tensions between familial obligations and personal interests or difficulties in interpersonal relationships (Hou et al., 2019; Zhang and Zhuo, 2024). This cultural perspective provides a sharper lens for understanding how threatened school belonging undermines self-consistency and congruence, thereby contributing to depressive symptoms.

### 5.3 The moderating effect of emotional intelligence

Additionally, this study reveals a “sensitivity double-edged sword” effect of emotional intelligence in social stress contexts: although high emotional intelligence typically confers enhanced capacities for emotional awareness and regulation, it may paradoxically amplify the adverse impact of stressors that threaten fundamental social needs and self-identity, such as low school belonging, on the core psychological construct of self-consistency and congruence. Specifically, emotional intelligence significantly moderated the first stage of the mediation model linking school belonging to depressive symptoms, such that students with higher emotional intelligence exhibited a stronger negative association between low school belonging and self-consistency and congruence, a pattern inconsistent with the commonly assumed protective role of emotional intelligence (He et al., 2013; Steare et al., 2024). This counterintuitive finding can be understood through multiple interrelated mechanisms. Individuals high in emotional intelligence demonstrate heightened sensitivity to and accurate interpretation of both internal and contextual emotional cues (Li et al., 2024). When experiencing low school belonging, they not only detect feelings of exclusion, disconnection, or social alienation (e.g., loneliness and sadness) more rapidly and precisely, but also engage in deeper appraisals of the discrepancy between these experiences and their fundamental need for relatedness. This refined cognitive-emotional processing may transform the external stressor of perceived low school belonging into an intensified internal conflict between self and environment, thereby amplifying its disruptive effect on self-consistency and congruence (He et al., 2013). In contrast, individuals low in emotional intelligence may exhibit blunted awareness of such relational threats and less integration of affective and cognitive information, which, albeit maladaptive in the long term, may temporarily buffer the subjective impact of belongingness deficits.

Although high emotional intelligence is typically linked to adaptive regulation strategies such as cognitive reappraisal (Li et al., 2024), its effects are contingent upon how emotional information is processed within specific socio-cultural contexts. When confronting chronic, identity-relevant stressors such as low school belonging, individuals with higher emotional intelligence may engage in a more intensive self-referential appraisal of their emotional experiences—particularly focusing on discrepancies between their perceived social standing and internalized standards of group acceptance. In cultural contexts like China, where interpersonal harmony and role-based expectations are emphasized, these standards are particularly salient. Consequently, heightened emotional awareness and metacognitive processing may foster an internally-focused, discrepancy-centered rumination. This amplifies the perceived gap between an ideal self (defined by relational inclusion) and actual experiences of social exclusion, thereby exacerbating self-incongruence in this context.

Conversely, individuals with lower emotional intelligence may be less prone to such deep reflection or more likely to employ distraction or avoidance strategies (Afzalur Rahim et al., 2002), which, though less adaptive in principle, may inadvertently attenuate the immediate psychological salience of the incongruence. Taken together, this culturally embedded processing pattern helps explain why emotional intelligence, rather than buffering stress, may strengthen the negative association between low school belonging and diminished self-consistency and congruence.

Therefore, fostering college students’ sense of school belonging and enhancing their emotion regulation capacity are crucial. In light of the specific mechanisms identified in this study, the following targeted intervention strategies are suggested. Firstly, structured belonging-building programs could be implemented by designing and offering low-threshold, theme-based group activities (e.g., orientation cohorts, academic peer circles) to create consistent positive social interactions that directly address the relational deficits undermining self-consistency and congruence (Hoang, 2025). Secondly, self-integration training could be provided by incorporating mindfulness and cognitive-behavioral techniques into workshops or courses, helping students non-judgmentally observe and reconcile discrepancies between their self-perceptions and social experiences (Sharma and Kumra, 2022), thereby cultivating a more harmonious and integrated self-concept. Thirdly, given that emotional intelligence can amplify distress in contexts of low belonging, emotion regulation skill modules could be integrated into curricula in a differentiated manner based on students’ levels of emotional intelligence (Li et al., 2024). For students with higher emotional intelligence, heightened emotional awareness can intensify their emotional responses to cues related to school belonging, thereby exacerbating declines in self-consistency and congruence. Interventions for this group should therefore emphasize redirecting emotional information toward cognitive reappraisal and problem-focused social behaviors. In contrast, students with lower emotional intelligence may benefit more from foundational training in emotion recognition and labeling to better understand social experiences and maintain self-consistency and congruence.

This strengthens the buffering role of emotional intelligence against the negative impact of low belonging. These mechanism-informed strategies may support universities in developing more precise and effective mental health promotion programs for Chinese college students.

## 6 Limitations

In summary, this study demonstrates that enhancing school belonging and emotional intelligence could promote self-consistency and congruence development among Chinese college students, ultimately alleviating depressive symptoms. However, three limitations should be acknowledged. Firstly, this study is based exclusively on cross-sectional self-report data, which precludes causal interpretation due to the absence of temporal precedence and is susceptible to methodological artifacts such as common method variance and socially desirable responding. To mitigate concerns regarding common-method variance and enhance construct validity, future research should integrate multi-method assessments. This includes behavioral paradigms (e.g., Cyberball) to index reactions to social exclusion as a proxy for belonging threat, and performance-based tasks of emotion recognition and regulation as complementary indicators of emotional intelligence. Incorporating physiological markers (e.g., cortisol levels or heart rate variability) could further provide objective indices of emotional and stress responses, helping to validate whether self-reported psychological distress in the context of low school belonging is accompanied by corresponding biological correlates. Additionally, ecological momentary assessment (EMA), implemented via mobile devices to repeatedly sample participants’ real-time experiences of belonging, affect, and self-perceptions in natural settings (e.g., classrooms or dormitories), could reduce retrospective bias and more accurately capture the dynamic interplay among key constructs. Complementing self-report data with such ecologically grounded approaches would also allow researchers to link momentary belonging experiences to observable behavioral indicators of engagement. In this regard, emerging evidence suggests that students’ subjective satisfaction alone may not adequately reflect their willingness to participate or remain engaged in institutional learning activities. For instance, post-pandemic research has reported substantial declines in enrollment and occupancy rates for online non-core courses, despite stable student satisfaction and even higher course completion rates (So et al., 2025). This pattern has been attributed to reduced opportunities for interpersonal interaction and belonging, highlighting the importance of relational factors beyond perceived instructional quality.

Building on these measurement and behavioral considerations, longitudinal research designs are needed to clarify the temporal ordering among school belonging, self-consistency and congruence, and depressive symptoms. Multi-wave assessments over extended periods can reveal how fluctuations in belonging prospectively predict changes in self-harmony and subsequent symptomatology. Randomized controlled trials—such as structured programs aimed at enhancing school belonging—offer a direct test of causal pathways by examining whether targeted manipulations produce expected downstream effects on psychological outcomes. Such designs not only validate theoretical models but also generate actionable evidence for culturally grounded mental health interventions. Together, the integration of multi-method, longitudinal, and intervention-based approaches promises a more comprehensive, dynamic, and culturally informed understanding of the mechanisms underlying psychological well-being in this population, thereby deepening both theoretical insight and practical impact.

Secondly, although this study employed convenience sampling to assess depressive symptoms among college students from Chongqing, Heilongjiang, and Liaoning provinces, the absence of recent normative data for Chinese college students’ depressive symptoms precluded accurate calculation and comparison of the detection rates for moderate to severe symptoms in our sample, potentially limiting cross-study comparability. Future research could establish nationally representative normative data for depressive symptoms among Chinese college students through stratified random sampling, with strata defined by institution type, geographic region, and grade. *A priori* sample size calculations based on expected effect sizes and desired statistical power should inform the sampling design. Such a normative framework would enhance the scientific rigor and cross-study comparability of prevalence estimates, thereby providing a more reliable, population-based benchmark for university mental health screening, risk monitoring, and program evaluation. Finally, although gender and grade were included as covariates, the present study did not examine whether the model varies across majors or grade levels, nor did it test additional potential moderators. Future research should assess the model’s invariance across subgroups (e.g., by major or year) and investigate other plausible moderating factors.

Furthermore, several additional avenues for future inquiry warrant consideration. One promising direction involves examining potential subgroup variations in the proposed model by assessing whether its structural associations differ meaningfully across academic disciplines, such as between STEM and humanities or social sciences, or across developmental stages of college life, including the transition-focused first year and the career-pressure-laden senior year, thereby informing more targeted psychological interventions. Another line of inquiry could incorporate culturally grounded constructs such as face consciousness and perceived relational mobility to test their moderating roles in the relationship between school belonging and mental health, thus enhancing the model’s cultural validity and contextual sensitivity within Chinese settings. In addition, the adoption of dynamic, process-oriented methodologies such as diary studies or experience sampling would enable intensive longitudinal assessment of daily fluctuations and reciprocal influences among key variables, offering valuable insight into short-term mechanisms and helping to address the causal limitations inherent in cross-sectional designs. Advancing research through these complementary approaches, which integrate macro-level norm development with micro-level mechanism exploration, may foster a more systematic and indigenous framework for mental health research among Chinese college students.

## 7 Conclusion

A decrease in Chinese college students’ sense of school belonging indirectly increases the likelihood of their depressive symptoms by reducing the level of self-consistency and congruence. During this process, emotional intelligence can exacerbate the negative impact caused by a low sense of school belonging. Therefore, colleges and universities in China can regard fostering college students’ sense of school belonging and improving their emotion regulation ability as effective approaches to reducing the risk of depression among college students.

## Data Availability

The raw data supporting the conclusions of this article will be made available by the authors, without undue reservation.
